# Supplementary material for: A naturally protective epitope of limited variability as an influenza vaccine target
Source: Nat Commun. 2018 Sep 21;9:3859. doi: 10.1038/s41467-018-06228-8 (PMC6155085; doi:10.1038/s41467-018-06228-8)
Supplement: Supplementary file 1 — Supplementary Information [file 41467_2018_6228_MOESM1_ESM.pdf]

Supplementary Information

A naturally protective epitope of limited variability as a novel influenza vaccine target

Thompson *et al.*

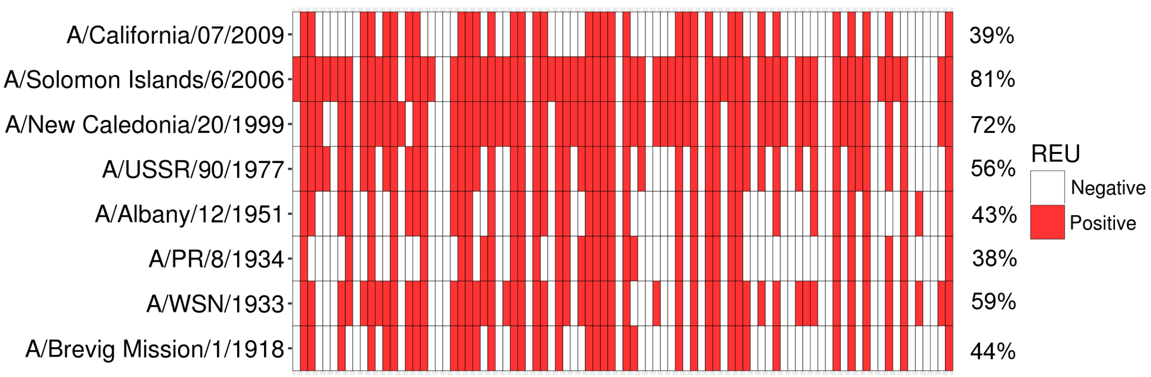

Supplementary Figure 1. ELISA analysis of sera from children. ELISA analysis of sera from children aged 6 to 12 years. The HA1 domain of a number of chronologically dispersed strains was used as an antigen. All ELISAs were standardised using positive sera. Samples were either allocated as ‘positive’ (red) or ‘negative’ (white) based on whether they fell into the linear range of the standard curve. The percentage of the samples that displayed reactivity to each strain is provided on the right hand side of the figure.

Supplementary Figure 2

a

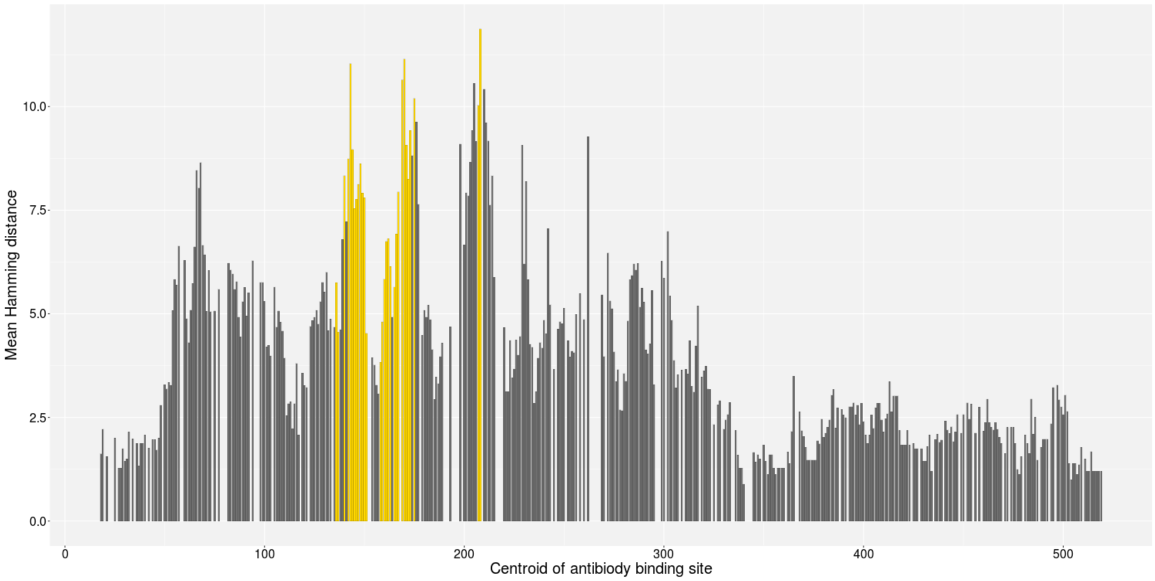

b

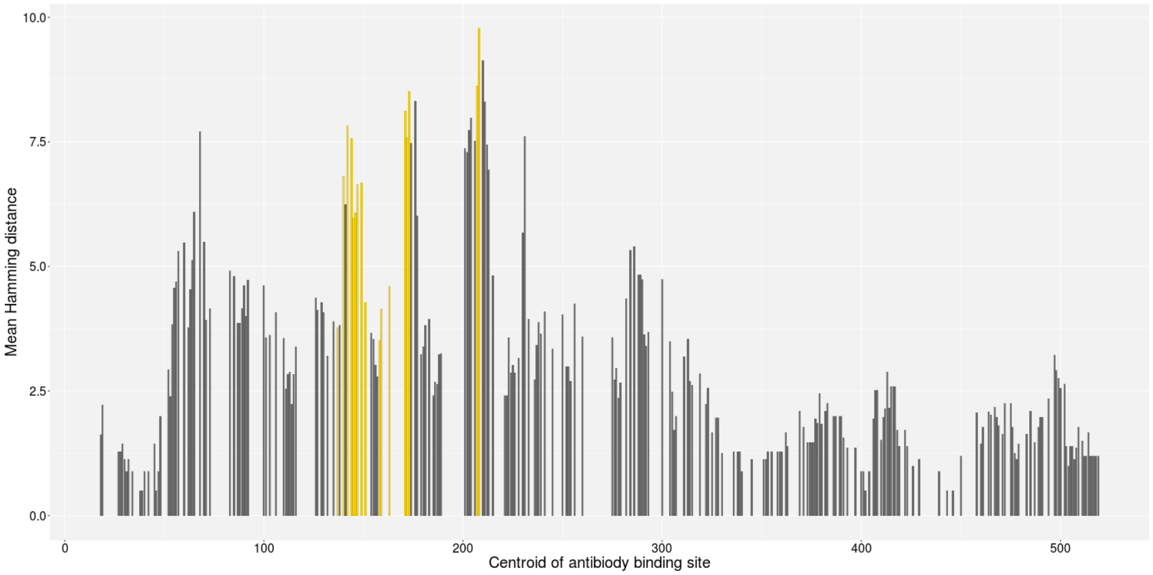

Supplementary Figure 2 (continued)

c

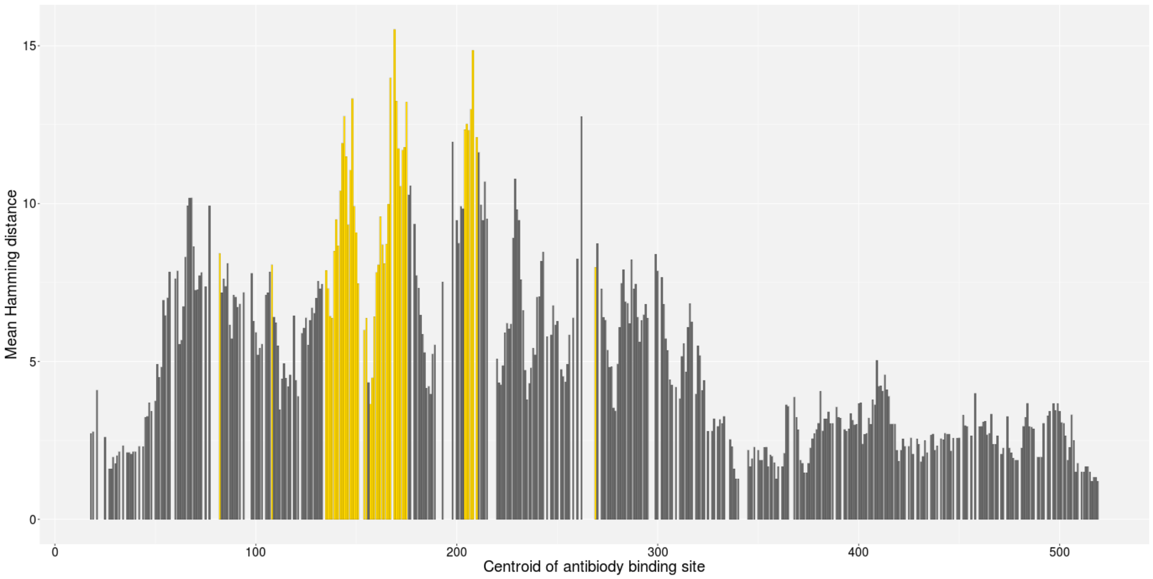

d

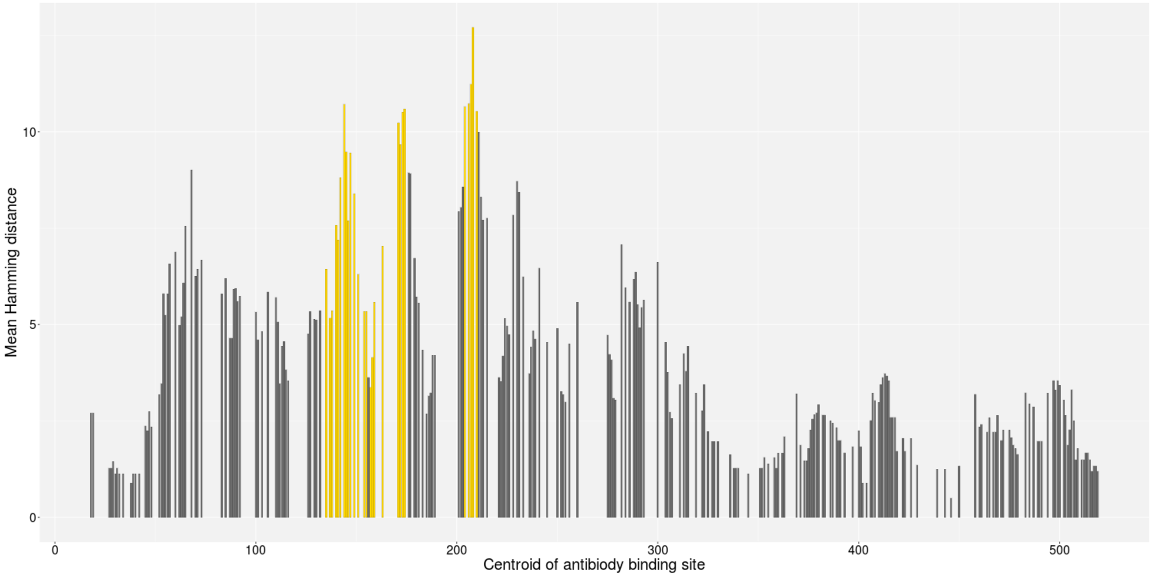

e

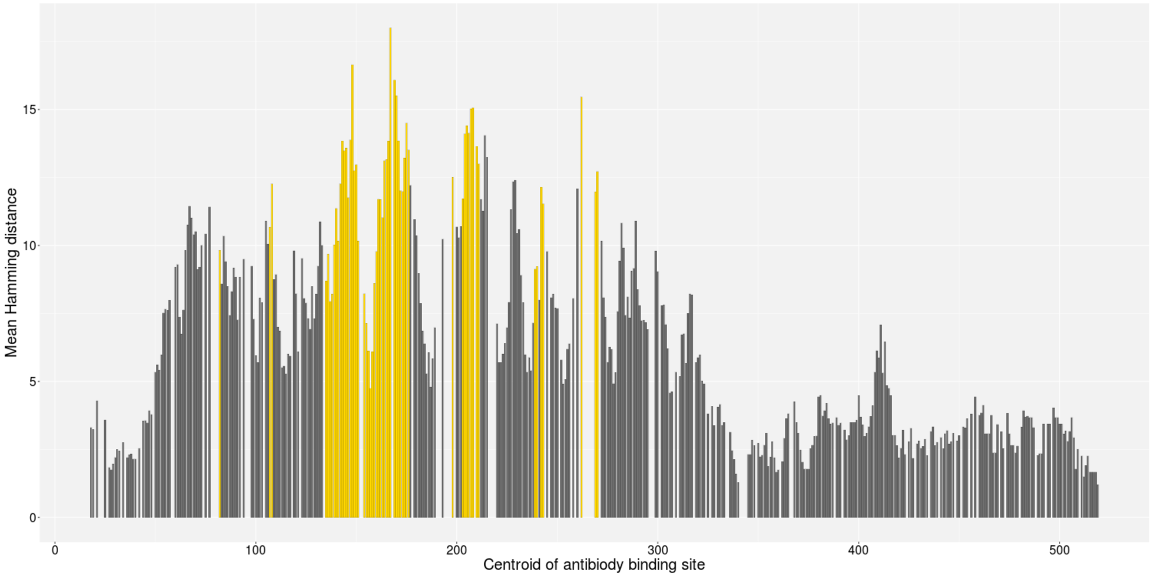

Mean Hamming distance
0
5
10
15
0
100
200
300
400
500
Centroid of antibiody binding site

f

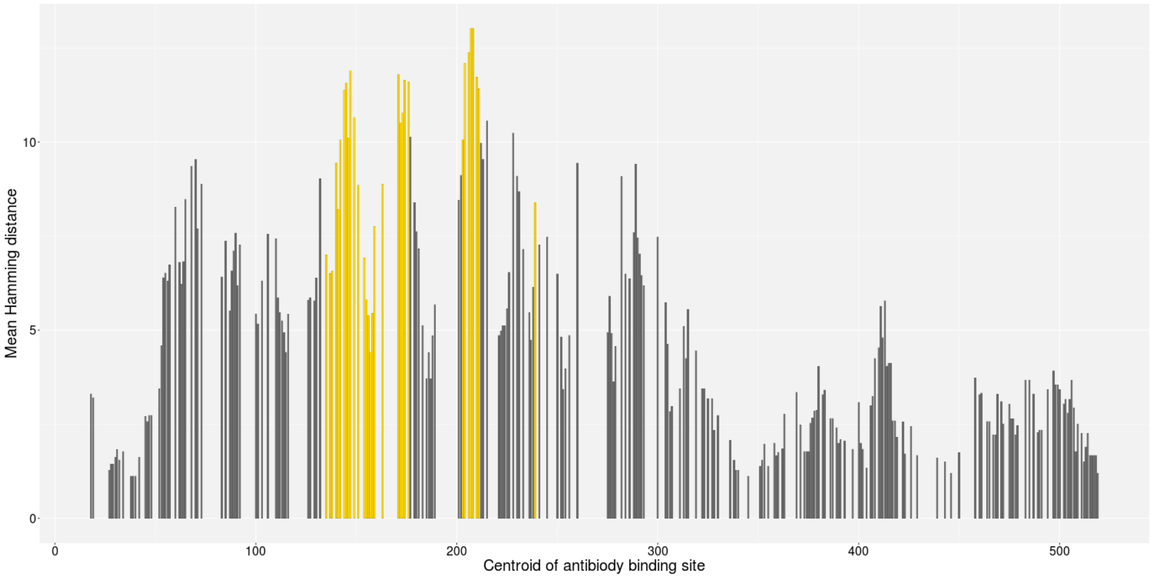

Mean Hamming distance
0
5
10
0
100
200
300
400
500
Centroid of antibiody binding site

g

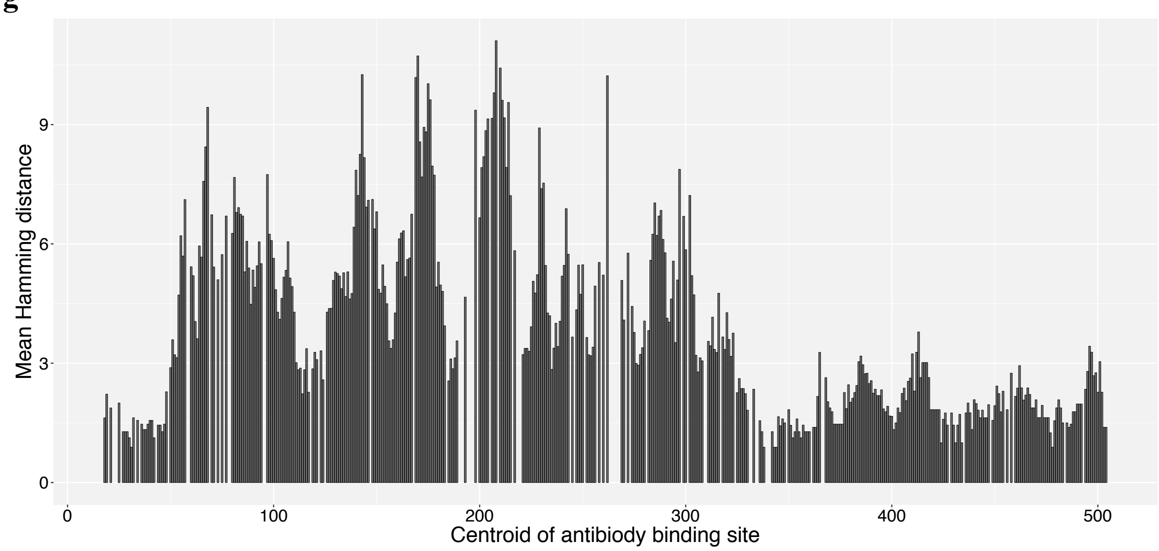
Mean Hamming distance
0
3
6
9
0
100
200
300
400
500
Centroid of antibody binding site

h

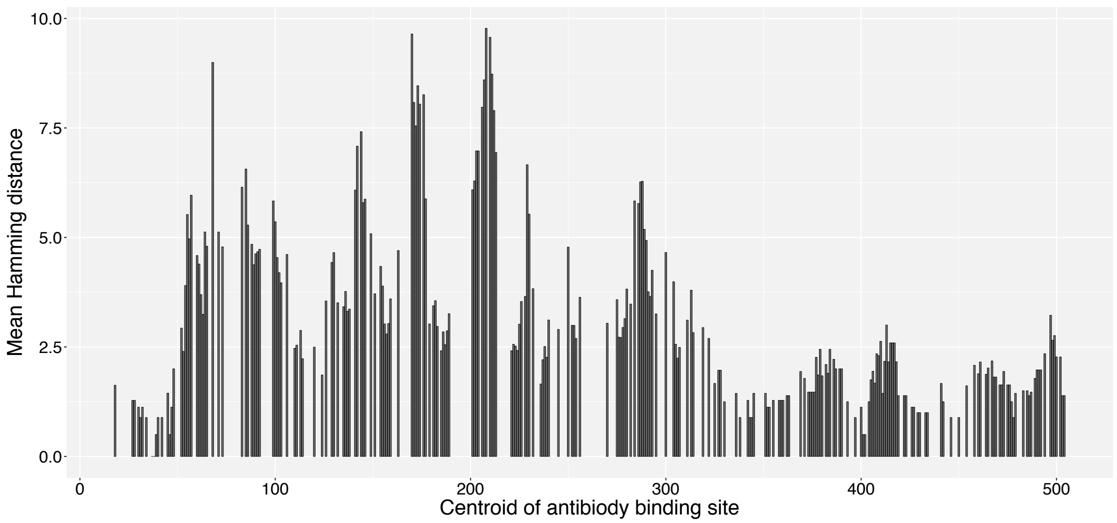
Mean Hamming distance
0.0
2.5
5.0
7.5
10.0
0
100
200
300
400
500
Centroid of antibiody binding site

i

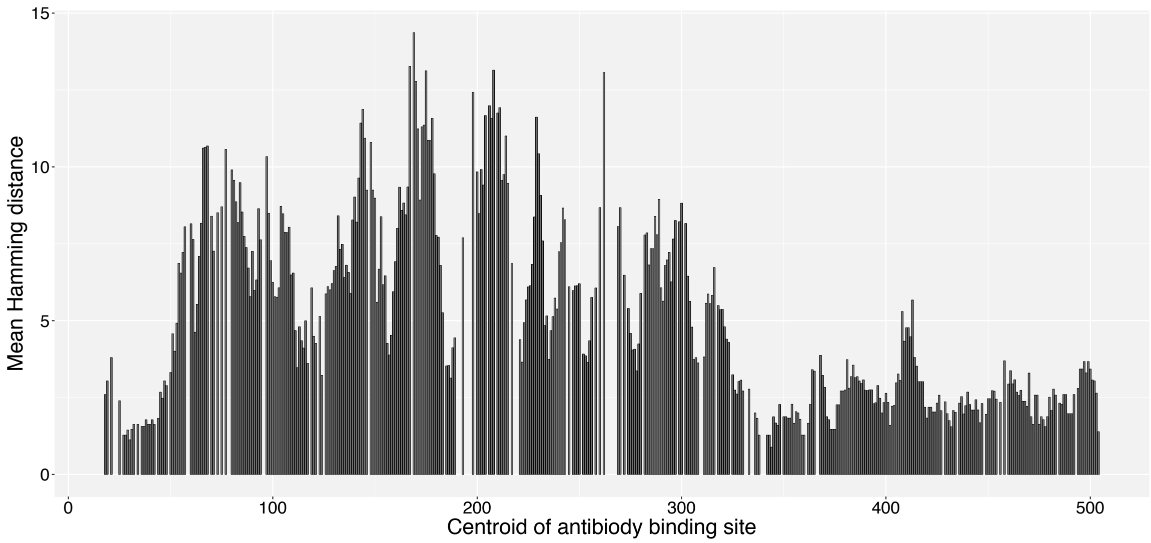
Mean Hamming distance
0
5
10
15
0
100
200
300
400
500
Centroid of antibiody binding site

Supplementary Figure 2 (continued)

j

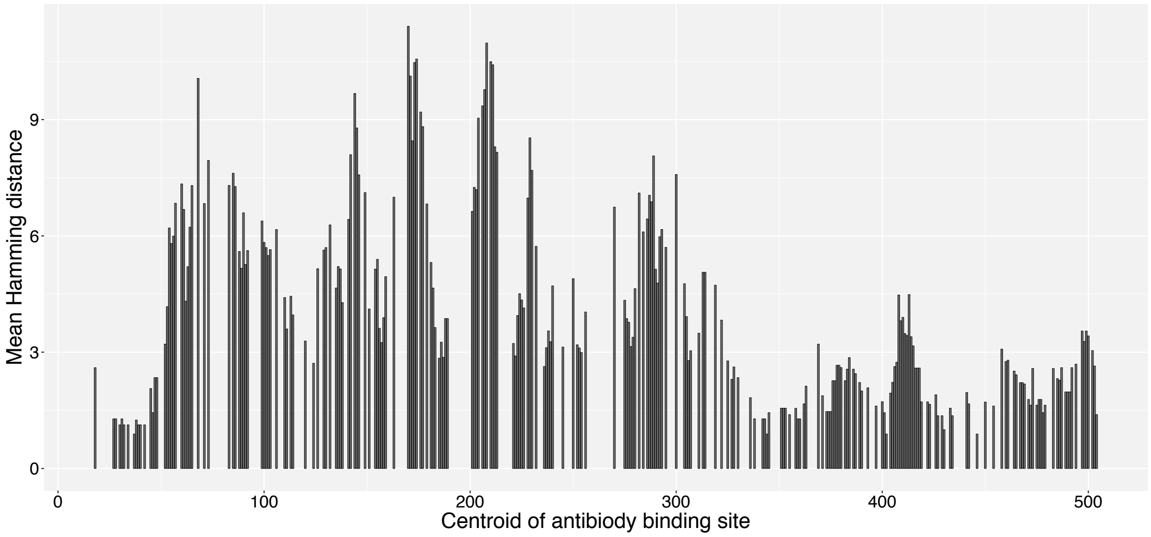

k

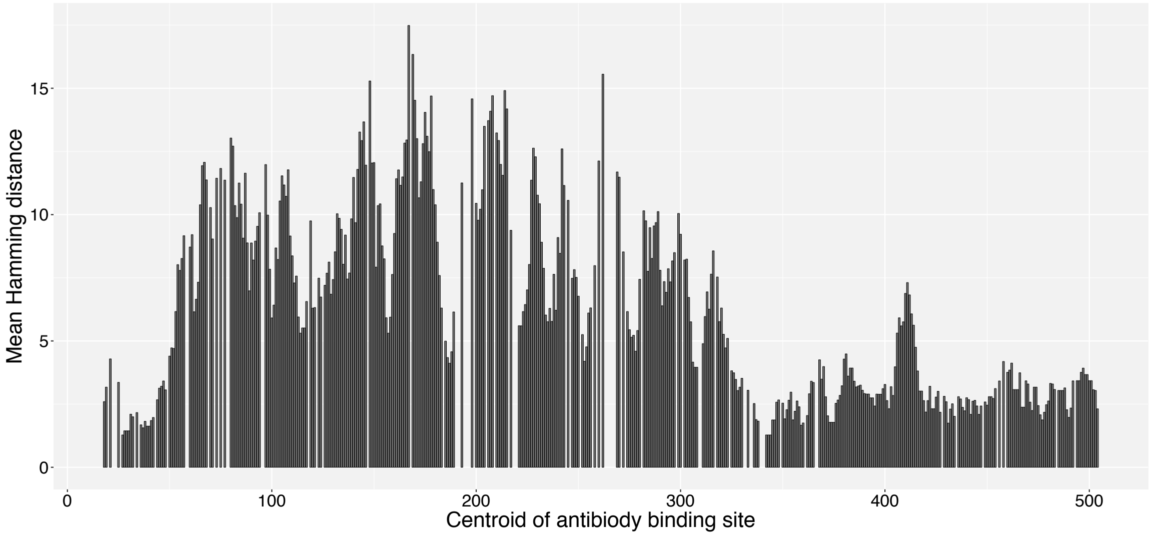

Supplementary Figure 2 (continued)

l

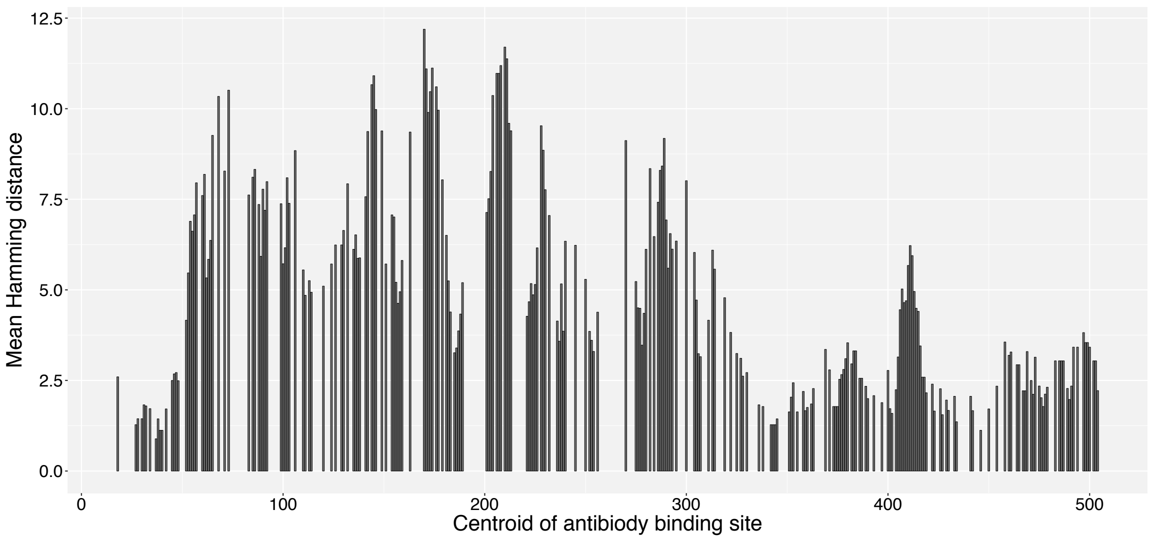

m

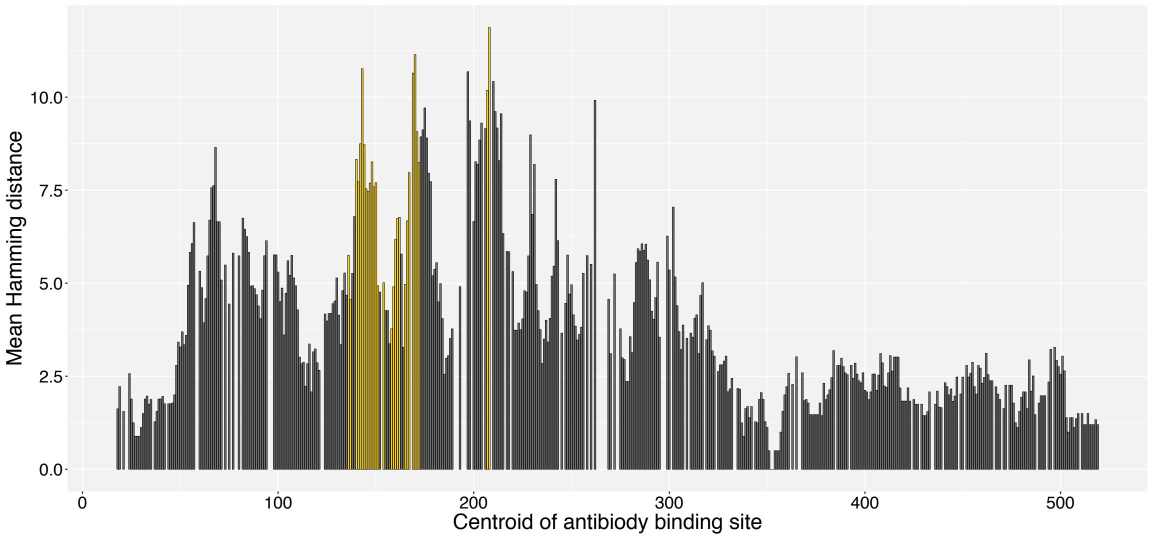

Supplementary Figure 2 (continued)

n

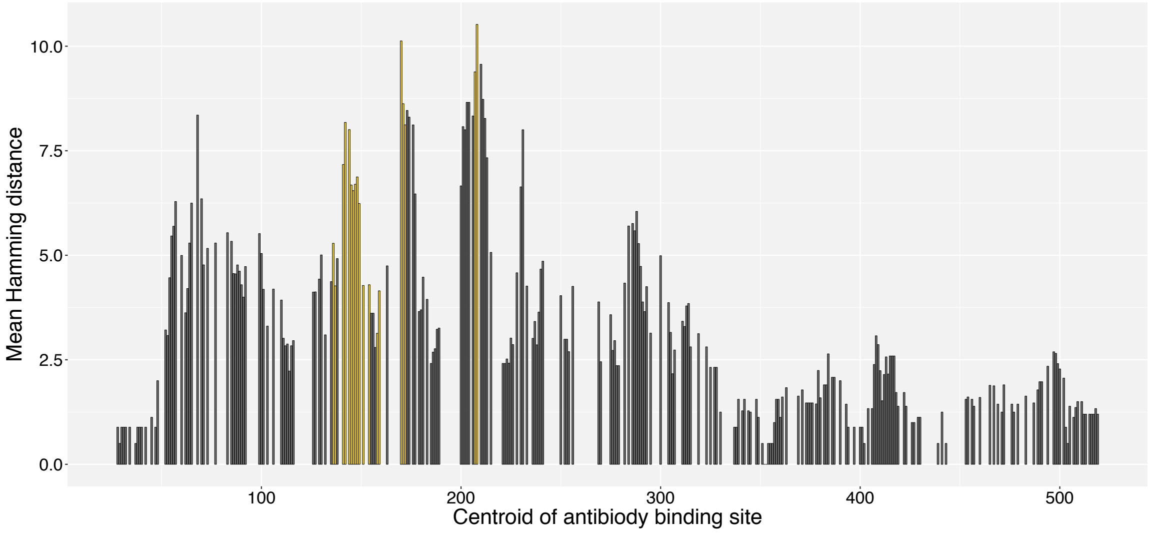

o

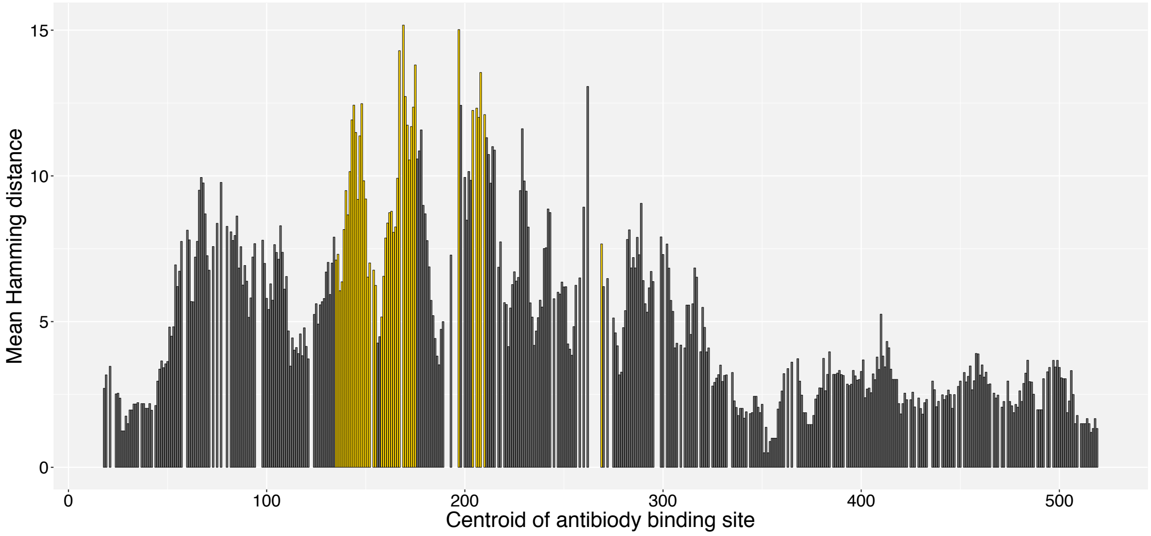

Supplementary Figure 2 (continued)

p

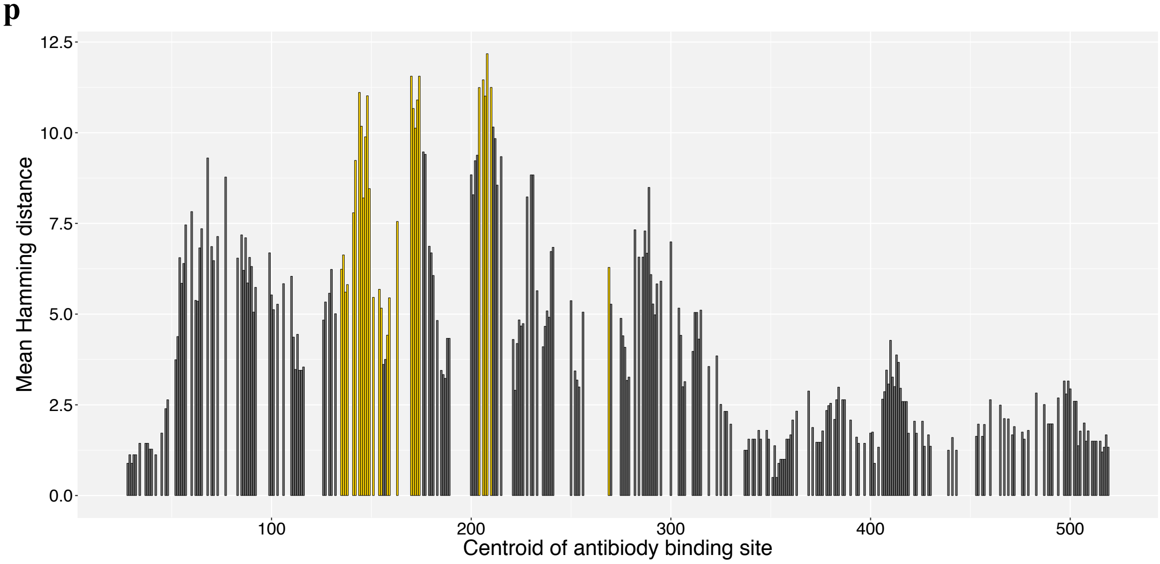

q

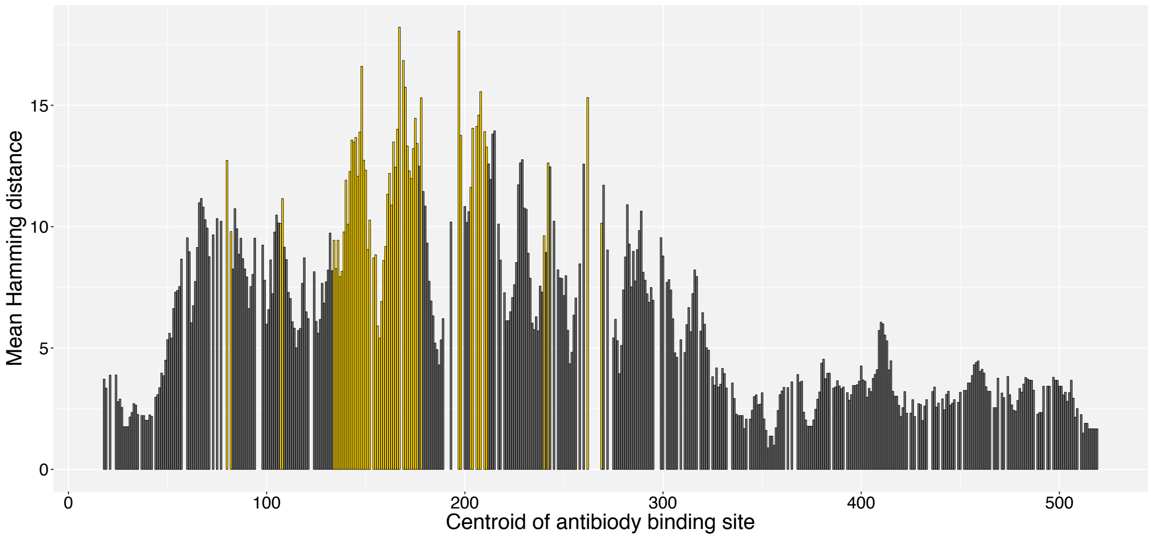

r

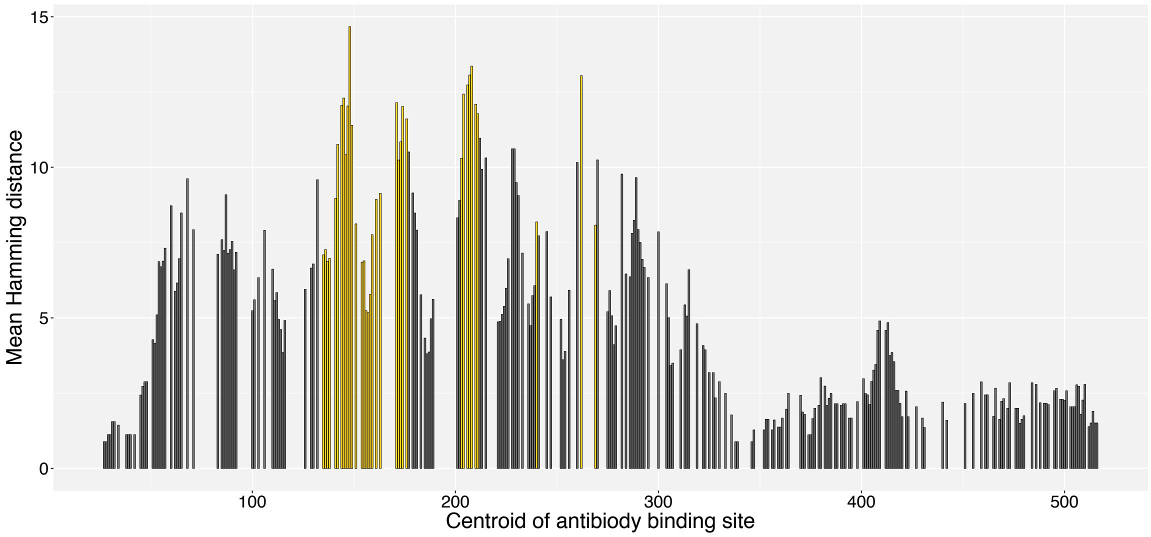

Supplementary Figure 2. Variation in antibody binding sites mapped onto the surface of various H1 HA crystal structures. Variability in antibody binding sites ABS were mapped to the crystal structure of A/California/04/2009 to determine the variability of each antibody binding site based on parameters of **a** 600 Å^2, >1% accessibility, **b** 600 Å^2, >10% accessibility, **c** 800 Å^2, >1 accessibility **d** 800 Å^2, >10% accessibility **e** 1000 Å^2, >1% accessibility **f** 1000 Å^2, >10% accessibility. Variability in antibody binding sites ABS were mapped to the crystal structure of A/PR/8/1934 to determine the variability of each antibody binding site based on parameters of **g** 600 Å^2, >1% accessibility, **h** 600 Å^2, >10% accessibility, **i** 800 Å^2, >1 accessibility, **j** 800 Å^2, >10% accessibility, **k** 1000 Å^2, >1% accessibility, **l** 1000 Å^2, >10% accessibility. Variability in antibody binding sites ABS were mapped to the crystal structure of A/Brevig Mission/1/1918 to determine the variability of each antibody binding site based on parameters of **m** 600 Å^2, >1% accessibility, **n** 600 Å^2, >10% accessibility, **o** 800 Å^2, >1 accessibility, **p** 800 Å^2, >10% accessibility, **q** 1000 Å^2, >1% accessibility, **r** 1000 Å^2, >10% accessibility. Accessibility is determined by the accessibility of an amino acid to a water molecule. The amino acid position at the centre of each centroid is used to denote the ABS. In each instance ABS including position 147 are highlighted in yellow. Similar analyses were performed for the A/Washington/05/2011 crystal structure producing a similar pattern to the A/California/04/2009 analysis data not shown. ABS highlighted in yellow include position 147. Equivalents of these ABS can be found in A/PR/8/1934 but these have not been highlighted as there is a deletion in position 147.

	146	147	148	149	151	154	155	156	157	158	159
2016	N	K	G	V	A	P	H	A	G	A	K
2015	N	K	G	V	A	P	H	A	G	A	K
2014	N	K	G	V	A	P	H	A	G	A	K
2013	N	K	G	V	A	P	H	A	G	A	K
2012	N	K	G	V	A	P	H	A	G	A	K
2011	N	K	G	V	A	P	H	A	G	A	K
2010	N	K	G	V	A	P	H	A	G	A	K
2009	N	K	G	V	A	P	H	A	G	A	K
2008	T	-	G	V	A	S	H	N	G	E	S
2007	T	-	G	V	A	S	H	N	G	E	S
2006	T	-	G	V	A	S	H	N	G	K	S
2005	T	-	G	V	A	S	H	N	G	K	S
2004	T	-	G	V	A	S	H	N	G	K	S
2003	T	-	G	V	A	S	H	N	G	K	S
2002	T	-	G	V	A	S	H	N	G	K	S
2001	T	-	G	V	A	S	H	N	G	K	S
2000	T	-	G	V	A	S	H	N	G	K	S
1999	T	-	G	V	A	S	H	N	G	K	S
1998	T	-	G	V	A	S	H	N	G	K	S
1996	T	K	G	V	A	S	H	N	G	K	S
1995	T	K	G	V	A	S	H	N	G	K	S
1993	T	K	G	V	A	S	H	N	G	K	S
1992	T	K	G	V	A	S	H	N	G	K	S
1991	T	K	G	V	A	S	H	N	G	K	S
1989	T	K	G	V	A	S	H	N	G	K	S
1987	T	K	G	V	A	S	H	K	G	K	S
1986	T	K	G	V	A	S	H	K	G	K	S
1985	T	R	G	V	A	S	H	K	G	K	S
1984	T	K	G	V	A	S	H	K	G	K	S
1983	T	K	G	V	A	S	H	K	G	K	S
1982	T	R	G	V	A	S	H	K	G	K	S
1981	T	R	G	V	A	S	H	K	G	K	C
1980	T	R	G	V	A	S	H	K	G	K	S
1979	T	R	G	V	A	S	H	K	G	K	S
1978	T	R	G	V	A	S	H	K	G	K	S
1977	T	R	G	V	A	S	H	K	G	K	S
1957	R	-	G	V	A	P	H	A	R	K	S
1954	T	R	G	V	A	S	H	A	K	K	S
1953	T	R	G	V	A	S	H	A	R	K	S
1951	T	R	G	V	A	S	H	A	K	K	S
1950	I/T	R	G	V	A	S	H	A	G	K	S
1949	T	R	G	V	A	S	H	K	G	K	S
1948	T	R	G	V	A	S	H	K	G	K	S
1947	T	R	G	V	A	S	H	A	G	K	S
1946	D/T/N	I	G	V	A	S	H	A	G	K	S
1945	T	R	G	V	A	S	H	A	G	K	S
1945	T	R	G	V	A	S	H	A	G	K	S
1943	A	R	G	V	A	S	H	A	G	K	S
1942	T	K	G	V	A	S	H	A	G	K	C
1940	N	I	G	V	A	S	H	A	G	K	S
1936	N	I	G	V	A	S	H	A	G	K	S
1935	T	K	G	V	A	S	H	A	G	K	S
1934	N	-	G	V	A	S	H	E	G	K	S
1933	L	K	G	V	A	S	H	G/R	G	K	S
1918	T	K	G	V	A	S	H	A	G	A	S

Supplementary Figure 3. Identification of the various conformations of a site of limited variability in the head domain of the H1 HA through structural bioinformatic analysis. Analysis of consensus sequences corresponding to the disrupted peptide sequence of the antibody binding site of lowest variability containing position 147 OREO in the >10%, 1000 Å^2 plot for A/California/4/2009.

a

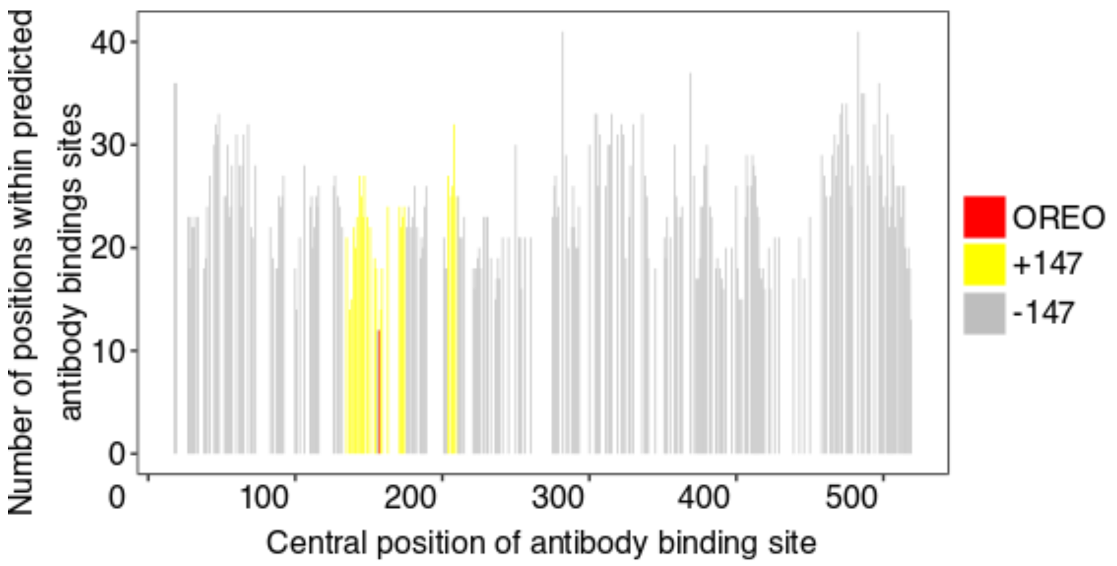

b

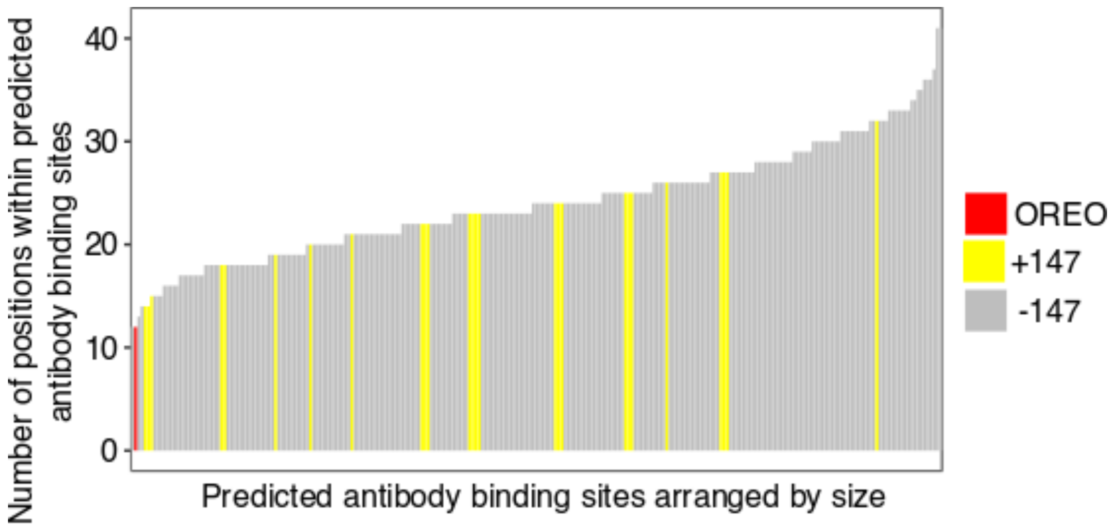

Supplementary Figure 4. **The number of residues in each predicted antibody binding site (ABS). a ABS distributed based on the location of the central amino acids in the ABS**. B. ABS arranged by size. OREO is one of the smallest ABS. OREO denotes the site referred to as 'OREO' in this paper and is shown in red. '+147' denotes sites containing position 147, which are shown in yellow. '-147' denotes sites that don't contain position 147, which are shown in grey.

a

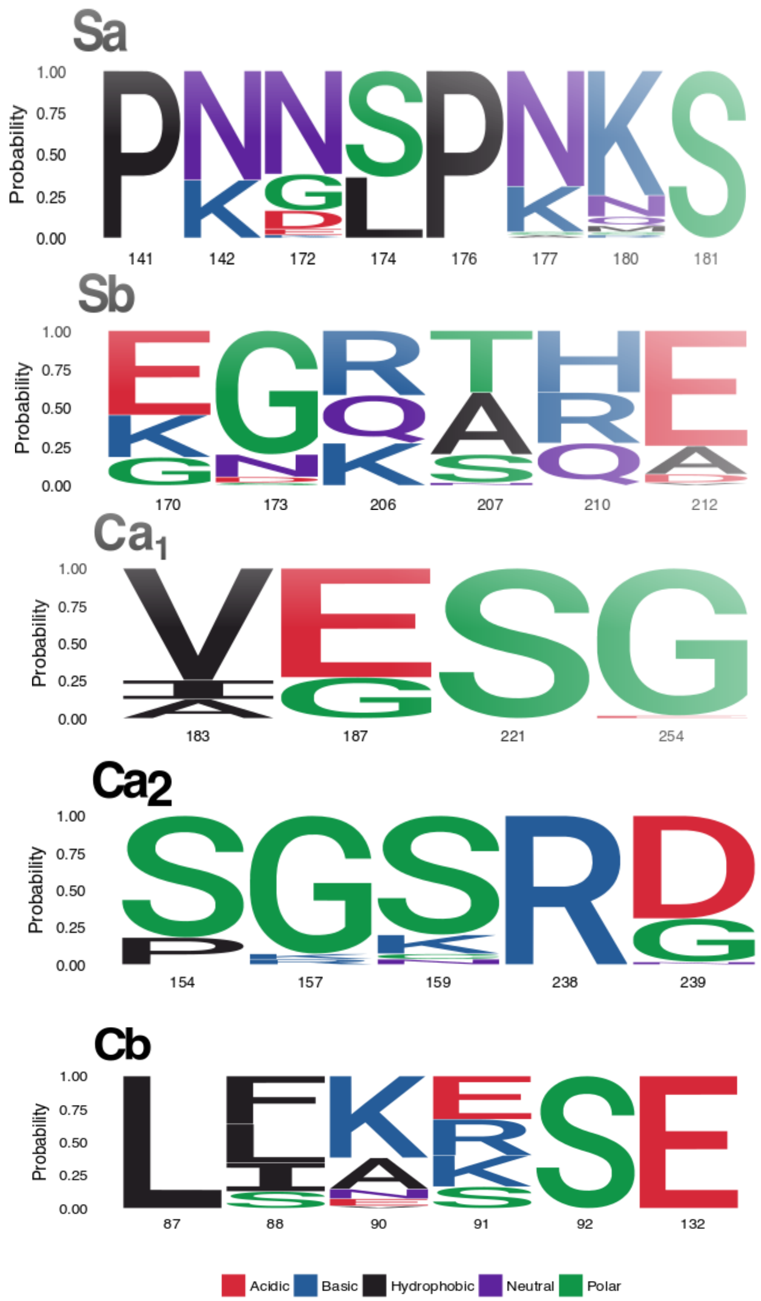
Sa
Probability
1.00
0.75
0.50
0.25
0.00
141
142
172
174
176
177
180
181
Sb
170
173
206
207
210
212
Ca1
183
187
221
254
Ca2
154
157
159
238
239
Cb
87
88
90
91
92
132
Acidic
Basic
Hydrophobic
Neutral
Polar

b

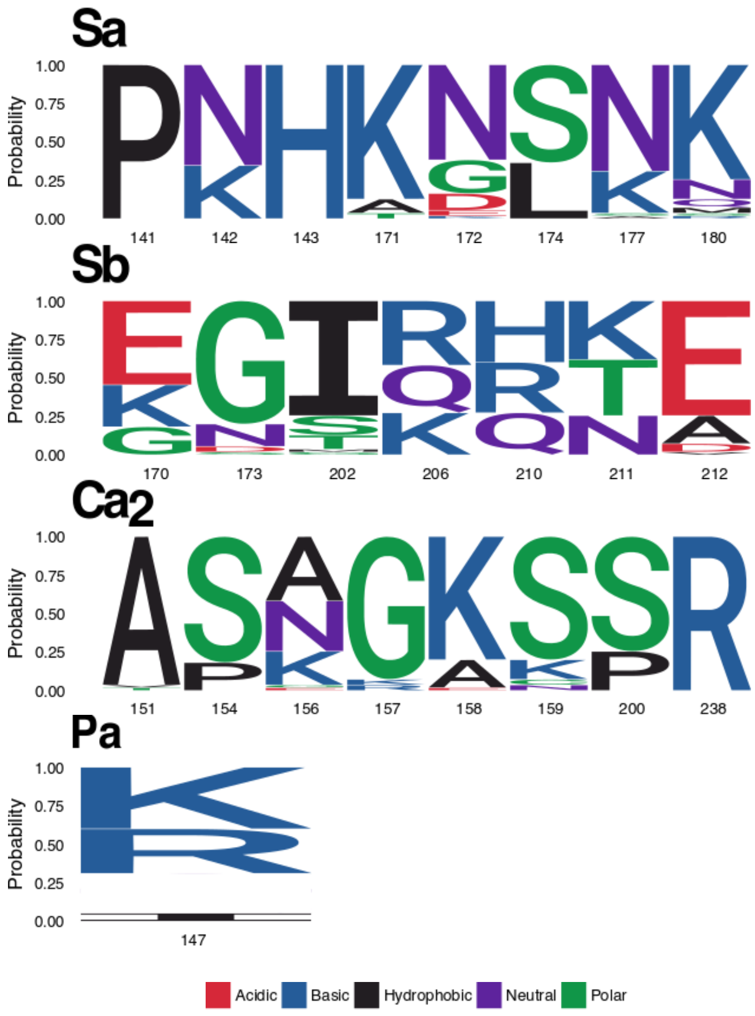

Supplementary Figure 5. Natural variation in antigenic sites defined by virus escape mutants. In **a** Caton et al 1982, and **b** Matsuzaki et al 2014[1,2]. The variation displayed in these figures is based on yearly consensus sequence.

a

Name	146	147	148	149	151	154	155	156	157	158	159
Red	T	Absent	G	V	A	S	H	N	G	K	S
H5	S	S	G	V	S	P	Y	Q	G	R	S
H6	S	T	G	V	R	P	Y	N	S	G	S
H11	G	A	G	V	A	K	F	G	S	S	N

b

Name	146	147	148	149	151	154	155	156	157	158	159
Green	T	R	G	V	A	S	H	K	G	K	S
H5	S	S	G	V	S	P	Y	Q	G	R	S
H6	S	T	G	V	R	P	Y	N	S	G	S
H11	G	A	G	V	A	K	F	G	S	S	N

c

Name	146	147	148	149	151	154	155	156	157	158	159
Orange	T	K	G	V	A	S	H	N	G	K	S
H5	S	S	G	V	S	P	Y	Q	G	R	S
H6	S	T	G	V	R	P	Y	N	S	G	S
H11	G	A	G	V	A	K	F	G	S	S	N

d

Name	146	147	148	149	151	154	155	156	157	158	159	163
Blue	N	K	G	V	A	P	H	A	G	A	K	K
H5	S	S	G	V	S	P	Y	Q	G	R	S	R
H6	S	T	G	V	R	P	Y	N	S	G	S	R
H11	G	A	G	V	A	K	F	G	S	S	N	R

e

Name	146	147	148	149	151	154	155	156	157	158	159	163
Pink	N	I	G	V	A	S	H	A	G	K	S	K
H5	S	S	G	V	S	P	Y	Q	G	R	S	R
H6	S	T	G	V	R	P	Y	N	S	G	S	R
H11	G	A	G	V	A	K	F	G	S	S	N	R

Supplementary Table 1. Sequences cloned into H6, H5 and H11 HAs. Amino acids changed in the H5, H6 and H11 backbones to the **a** red conformation 2006 H1 HA sequence, **b** green conformation (1977 sequence), **c** orange conformation (1995 sequence), **d** blue conformation (2009 sequence) and **e** pink conformation (1940 sequence).

Supplementary Table 2: Accession numbers pre-2009 pandemic

AAD172291918 AAA92280 ABF21278 ACV49534 AAM75158 ABD77675 ABO21709 ACF41834 AFM68954 AFM71846 AGU93019 CAA24272 ABD62781 ABN59412 ABO38384 AEM60005 ABO383511936 ABI20826 ABD62843 ABD79101 ABO38054 ABO38373 ABP493271945 ABD79112 ACV49556 AGQ47776 AAA67338 ABD77807 AEM23889 AGQ47764 ABN594011948 ABN59434 ADT78929 ABD61735 ABP49316 ADT78868 ABP49481 ABQ01311 ABQ44471 ADT78936 ADT79108 AGQ47811 ADT789081953 AAA43171 ABD60966 ABD15258 AFV53260 ABD60933 ABD60944 ABD95350 ABO44134 ABF47693 ABF47715 ABN59423 ABO32981 ABO32992 ABO38065 ABP49338 ABP49448 ABY81349 AAA43172 ABQ01322 ABW36311 ABF47748 ABO33006 ABO38362 ABO522581981 ABD77818 ABD95339 ABO52797 ABF47759 ABG26824 ABG88333 ABG88344 ABI92302 ABK40546 ABK40590 ABL67264 ABN50900 ABO33025 ABO38406 ABP49349 CAA910801985 ABF21274 ABM22246 ABO38395 ABO44123 ABP49360 ABN50928 ABN50940 ABQ44416 ACK99443 ACL12261 BAA02768 BAA02769 AAP34322 ABD60955 ABF21276 ACF41933 AFO64835 AFQ90533 AFO64846 AFQ90528 AAK70449 AAK70450 AAP34323 ABE11867 ABE11878 ABE11889 ABE11900 ABE11922 ABE11942 ABE12032 AAP60036 AAP60037 ABD59847 ABF47649 ABF47660 ABG47829 ABI20848 ABI21519 ABI21530 ABI21541 ABD59848 AFJ74459 AFJ78298 AFJ78364 AFO64879 AFO64890 AAK70451 AAK70452 AAK70453 AAK70454 AAK70456 AFJ78375 AFO64901 AFO65016 AAP34324 ABK40006 ACD37430 ACR15304 AFO64802 AAX56530 ABA08486 ABD60856 ABD60867 ABD60900 ABD61518 ABD77917 ABD77928 ABD77972 ABD94976 AAZ38627 AAZ79604 AAZ83299 ABA08519 ABA12715 ABA18037 ABA42236 ABA42575 ABA43189 ABA87045 AAZ83253 ABA87080 ABB51962 ABB82216 ACA96508 ACR15216 ACR15315 ACR15326 ADY04727 AFO66191 ABA08475 ABA12696 ABA12729 ABB03123 ABB53740 ABB82205 ABI96108 AEX33420 AEX34484 AFJ78386 ABC42750 ABW81513 ABW81518 AEP32457 AFJ78419 AFO66235 AFO66246 CDR50099 ABI21189 ABI21222 ABI21233 ABJ09184 ABJ16664 ABK40689 ABO32678 ABP49393 ABW81514 ABK79959 ABS71664 ABS71666 ABS71667 ABS71668 ABS71669 ABS71670 ABS71672 ABS71673 ABU50566 ABU50572 ABV29579 ABV29601 ABV29612 ABV29634 ABV29645 ABV29656 ABV29678 ABV29689 ABV29733 ACB11768 ACB11769 ACB11777 ACB11782 ACB11786 ACB11793 ACD47247 ACD47249 ACD47253 ACD47256

Supplementary Table 3: Accession numbers post-2009 pandemic:

AQN74335 ANM94671 AND61374 APF45854 APF45877 ANM87589 ANH71227 AMP44845 APR73864 ANM92713 AKS48055 AKS48056 AKS48057 ALH26300 ALH26362 ALH26389 ALH26394 ALH26424 ALH26482 ALH26545 AHJ57568 AHJ57578 AIM39597 ALJ53489 AHJ57581 AHV83886 AHV83823 AID47915 AKT08491 AIP92725 AGQ16244 AHJ57559 AHG96342 AGL09784 AHV83829 AGV29162 AGV28861 AGO02703 AHG96318 AIL94400 AFB18654 AFE02927 AFH41323 AFK25601 AFK25605 AFL56683 AFR68163 AFU09862 AGB08295 AGB08296 AGK72370 AFN19978 AFC98277 AEH59397 AHC68973 AGK72443 BAM37909 ADV58939 AEJ83843 BAM37823 ADW95319 AJE62470 AJS16287 AJE62461 ADO79927 AEK21426 AEH59365 ADF27885 AEI86844 ADV19292 ACP41934 ACQ84467 ACP44147 AIJ10864 ADD97928 ACY46333 ADM14589 ACR08541 AFJ78671 AIL90334

Supplementary References

1. Caton, A. J., Brownlee, G. G., Yewdell, J. W. & Gerhard, W. The antigenic structure of the influenza virus A/PR/8/34 hemagglutinin (H1 subtype). *Cell* **31,** 417–427 (1982).

2. Matsuzaki, Y. *et al.* Epitope Mapping of the Hemagglutinin Molecule of A/(H1N1)pdm09 Influenza Virus by Using Monoclonal Antibody Escape Mutants. *J. Virol.* **88,** 12364–12373 (2014).
